# Trends of Cocaine Use and Manifestations in Hospitalized Patients: A Cross-Sectional Study

**DOI:** 10.7759/cureus.22090

**Published:** 2022-02-10

**Authors:** Karthik Gangu, Aniesh Bobba, Sanket D Basida, Sindhu Avula, Harleen Chela, Simranjit Singh

**Affiliations:** 1 Department of Internal Medicine, University of Missouri, Columbia, USA; 2 Department of Internal Medicine, John H. Stroger, Jr. Hospital of Cook County, Ohio, USA; 3 Graduate Student, Pandit Deendayal Upadhyay Medical College, Rajkot, IND; 4 Department of Cardiovascular Medicine, University of Kansas Medical Center, Kansas, USA; 5 Internal Medicine, Indiana University School of Medicine, Indianapolis, USA

**Keywords:** financial burden, trend, abuse, opioid, stimulant, hallucinogen, alcohol, sedative, marijuana, cocaine

## Abstract

Objective

About 41 million people aged ≥18 years reported lifetime use of cocaine, and 5.4 million people reported having used cocaine in 2019. We aim to identify trends of cocaine use, manifestations, concomitant drug use, and financial burden on health care among hospitalized patients.

Methods

We utilized National Inpatient Sample from years 2006-2018. Patients with age ≥18 years, admitted to the hospital with a diagnosis of cocaine abuse, dependence, poisoning, or unspecified cocaine use were included in the study. We used ICD-9 Clinical Modification (CM) and ICD-10-CM codes to retrieve patient samples and comorbid conditions. The primary outcome was the trend in cocaine use among hospitalized patients from the year 2006 to 2018. Cochran-Mantel-Haenszel test was used to assess the significance of trends.

Results

In the year 2006, the prevalence of cocaine abuse among hospitalized patients was 10,751 per million with an initial decline to 7,451 per million in 2012 and a subsequent increase to 11,891 per million hospitalized patients in 2018 with p =0.01. The majority of patients admitted were older than 50 years (43.27%), and a greater percentage of patients were males. All ethnicities showed a rising trend in the use of cocaine except for Native Americans. Cardiovascular effects, neuropsychiatric and infectious manifestations in hospitalized patients with cocaine abuse showed a consistent increase from year 2006 to 2018 with p <0.001.

Conclusions

There is a recent uptrend in cocaine use among hospital admissions in the US from 2006 to 2018 with an increased rate of systemic manifestations. This highlights the impact of cocaine use on the health system and the dire need to address this growing problem.

## Introduction

Cocaine is a weakly alkaline compound first isolated and named as erythroxyline by Friedrich Gaedcke in 1855 [1]. The global street value of cocaine was estimated to be $70.5 billion in 2003, of which 62% was from North America, followed by Europe (26%) [2]. In the 1800s, cocaine was freely sold in grocery stores, salons, and drug stores [3]. However, it was then classified as an illegal drug in 1915 in the United States (US) when Congress passed the Harrison Anti-Narcotics act [4]. The first epidemic of cocaine use in the US was seen in the 19th century [3].

According to the reports published by Substance Abuse and Mental Health Services Administration (SAMHSA) 2020, in 2019, over 41 million people aged 18 years or more reported lifetime use of cocaine, and 5.4 million people reported having used cocaine in the past year [5]. In 2017, emergency departments reported 70,237 deaths due to drug overdose, a 9.6% rate increase compared to 2016 [6]. Of these, cocaine contributed to a 32.9% increase in deaths compared to the previous year [6]. Long-term use of cocaine adversely affects the cardiovascular, neurological, and psychological health of users [7, 8]. It also increases pregnancy-related complications, exacerbates mental health disorders and overall mortality [8, 9].

Cocaine can be consumed through inhalation (snorting), oral, and intravenous forms [10]. Inhaled cocaine reaches the nasal mucosa, which absorbs the drug and provides entry into the bloodstream [10]. Inhaled forms are also associated with lung injuries and can cause blood concentrations as high as injectable forms [10]. Cocaine is also rubbed on gums, which is then systemically absorbed [10]. The powdered form of cocaine is mixed with water and injected directly into the bloodstream to experience faster drug effects. Due to these rapid euphoric effects, cocaine abuse became very popular during the 1980s [10].

There is limited data regarding cocaine abuse and dependency in hospitalized patients and its impact on mortality, morbidity, associated medical conditions, and prevalence of other substance abuse disorders. We hypothesized that the prevalence of cocaine use was increasing among hospitalized patients and associated medical diseases over the last decade. This study aimed to identify the prevalence and trend of cocaine use in hospitalized patients using the national inpatient sample of 2006 to 2018.

## Materials and methods

Data source

We utilized the National inpatient sample (NIS) from Agency for Healthcare Research and Quality (AHRQ) [11] from 2006 to 2018.

Inclusion and exclusion criteria

All patients with age ≥18 years, admitted to the hospital with a principal diagnosis or secondary diagnosis of cocaine-related disorders (cocaine abuse/ cocaine dependence/ cocaine use unspecified/ cocaine poisoning) were included in the study. We used ICD-9 Clinical Modification (CM) and ICD-10-CM codes to retrieve patient samples and comorbid conditions. A detailed code summary is provided in supplementary table 1. Patients aged <18 years with elective admission were excluded from the study. For the reader’s convenience, any “drug use” represented in this manuscript refers to abuse, dependency, unspecified, and poisoning except for in-remission.

Covariates

The NIS data sample contains data regarding in-hospital outcomes, procedures, and other discharge-related information. Variables were divided as follows into patient level, hospital level, and illness severity.

a. Patient-level: Age, race, sex, comorbidities, insurance status, income in patient’s zip code, disposition.

b. Hospital level: Location, teaching status, bed size, region.

c. Illness severity: Length of stay (LOS), mortality, hospitalization cost, Charlson comorbidity score (CCS).

Comorbidity

Comorbidities associated with cocaine use were sorted into cardiovascular, infectious disease, pulmonary, psychiatry, neurological systems as below. We used diagnostic codes to identify these comorbidities (Supplementary table).

a. Cardiovascular: Acute myocardial infarction, hypertension, arrhythmia, cardiomyopathy, heart failure, peripheral vascular disease.

b. Neurological: Cerebrovascular disease, hemiplegia/paraplegia, seizure.

c. Pulmonary: Asthma

d. Infectious disease: HIV, hepatitis.

e. Psychiatry: Psychosis, depression.

Study outcomes

The primary outcome was trends in cocaine use among hospitalized patients from 2006 to 2018. Secondary outcomes were (a) prevalence and trend of cocaine-associated comorbidities, (b) top 10 reasons for admission, (c) rates of concomitant drug use with cocaine, and impact on in-hospital mortality and (d) financial burden on healthcare and resource utilization.

Statistical methods

STATA 17 (StataCorp LLC, College Station, Tx) was utilized for statistical analysis. The weighted sample was around 35 million discharges for each calendar year. For ease of calculation, we used even-numbered years starting from 2006 to 2018. Trends were calculated utilizing the trend weights provided with the data sample, and rates were expressed per million admissions for that calendar year. Cochran-Mantel-Haenszel test was used to assess the significance of trends. Multivariate regression analysis was used to analyze the effect of concomitant drug use on mortality. Total hospitalization charge for each year was accounted for inflation using the consumer price index for the year 2018. Predictive margins were used to account for changing demographics, age with time, and impact on LOS and hospitalization cost.

## Results

Patient characteristics and trend analysis

**Figure 1:**
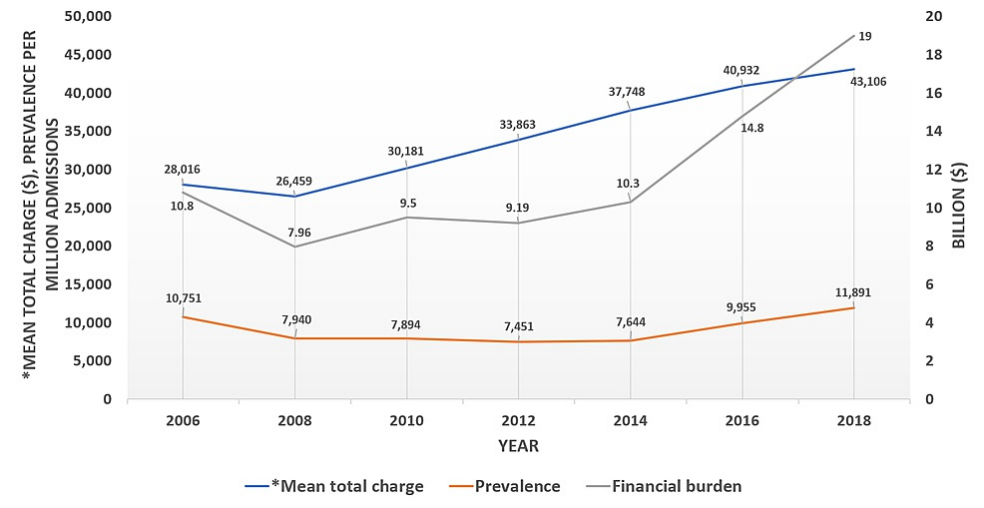
Cocaine use trends and its financial burden on healthcare Lt Y-axis: Prevalence of cocaine use among hospitalized patients (red line) – represented per million discharges for that year. Mean total charge of hospitalization was adjusted for age, sex, race, charlson comorbidity index, hospital bed size, teaching status, location and insurance. Rt Y-axis: Total financial burden due to hospitalization from cocaine use for that financial year (grey line, expressed in billion $).

Out of 261.38 million admissions, 2,368,886 had cocaine use in the years 2006 to 2018. In the year 2018, the mean age was 44.86, and females were 35.09%. The trend analysis of cocaine use in hospital admissions showed an initial decrease from 2006 (424,129) to 2014 (270,790) and an increase from 2016 (355,095) to 2018 (422,481) (Figure 1).

Patients aged 30 to 49 constituted 63.49% of admissions in 2006, with a steady decline to 42.17% in 2018. Patients over 50 comprised 21.2% of admissions in 2006, with a steady increase to 43.27% in 2018. Female sex showed a decreasing trend (36.45% to 35.09%, p=0.001) of admissions while an increasing trend was noted in males (41.85% to 46.64%, p=0.001) and ethnicities except for Native Americans. In 2018, 73.5% of patients were either Medicare or Medicaid insured, rising from a combined 60.49% in 2006 (p<0.001). The non-regular disposition, which includes discharge to a nursing home with home health or left against medical advice constituted approximately 25% of all discharges with a significant increasing trend, while regular disposition constituted the rest with a decreasing trend. The corresponding data is shown in Table 1. 

**Table 1:** Characteristics of patients admitted to hospital with Cocaine use *Adj for Age, sex, race, CCS, hospital bed size, teaching status, location and insurance. ¥Cocaine abuse, dependency, poisoning, unspecified use among admitted patients. ¤Calculated by dividing number of admissions with cocaine use with total number of admissions for that year. AMA=Left against medical advice; SNF=Discharge to long term acute care facility/skilled nursing home/rehab transfer; LOS=length of stay

Variable	2006	2008	2010	2012	2014	2016	2018	p-Trend	Trend Direction
No of admissions with cocaine use (N=)^¥^	424,129	316,710	307,856	271,825	270,790	355,095	422,481	-	-
Prevalence per million admissions^¤^	10,751	7,940	7,894	7,451	7,644	9,955	11,891	0.01	Increasing
Total admissions (millions)	39.45	39.89	39	36.48	35.36	35.67	35.53	-	-
Age (years)									
18-29	15.28%	15.4%	12.52%	12.78%	12.91%	15.53%	14.54%	0.6	No trend
30-49	63.49%	59.36%	54.73%	50.11%	45.69%	44.05%	42.17%	<0.001	Decreasing
50-69	20.84%	24.83%	32.03%	36.35%	40.32%	39.21%	41.59%	<0.001	Increasing
≥70	0.36%	0.38%	0.7%	0.75%	1.06%	1.2%	1.68%	<0.001	Increasing
Mean Age (SD)									
Female	39.01(10.07)	39.99(10.69)	41.79(10.7)	42.13(11.46)	42.87(11.87)	42.57(12.44)	43.09(12.81)	<0.001	Increasing
Male	41.85(10.56)	42.78(10.9)	44.7(11.03)	45.24(11.59)	45.97(12.09)	45.6(12.68)	46.64(13.01)	<0.001	Increasing
Sex (Female)	36.45%	36.85%	35.48%	35.46%	35.69%	35.21%	35.09%	0.011	Decreasing
RACE									
Caucasian	39.73%	43%	34.52%	37.52%	37.17%	41.24%	40.88%	<0.001	Increasing
African American	45.19%	43.16%	52.68%	47.01%	48.35%	44.02%	43.95%	<0.001	Increasing
Hispanic	11.52%	10.71%	9.22%	10.96%	10.19%	10.73%	11.05%	0.04	Increasing
Asian or Pacific Islander	0.32%	0.47%	0.39%	0.43%	0.52%	0.56%	0.59%	<0.001	Increasing
Native American	0.89%	0.51%	0.44%	0.48%	0.5%	0.54v	0.52%	0.83	No trend
Other	2.32%	2.11%	2.71%	3.57%	3.24%	2.88%	2.97%	0.004	Increasing
Income in patients zip code									
0-25^th^ percentile	48.37%	47.43%	52.28%	51.79%	52.08%	50.85%	48.5%	0.15	No trend
26-50^th^ percentile	22.79%	24.45%	21.82%	21.36%	22.15%	21.01%	23.13%	0.84	No trend
51-75^th^ percentile	17.27%	16.59%	16.44%	16.08%	15.05%	16.69%	16.72%	0.96	No trend
>76^th^ percentile	11.55%	11.51%	9.44%	10.75%	10.7%	11.43%	11.62%	0.5	No trend
Insurance									
Medicare	18.05%	19.21%	19.7%	21.89%	21.71%	20.87%	21.7%	<0.001	Increasing
Medicaid	41.99%	40.96%	44.9%	45.39%	50.8%	51.55%	51.81%	<0.001	Increasing
Private	15.39%	17.85%	11.99%	12.82%	12.93%	14.03%	13.1%	0.02	Decreasing
Self-pay	24.56%	21.96%	23.38%	19.87%	14.55%	13.53%	13.38%	<0.001	Decreasing
Hospital Region									
Northeast	28.33%	27.53%	32.45%	30.59%	28.79%	27.64%	27.38%	0.8	No trend
Midwest	22.57%	21.7%	18.92%	20.79%	20.69%	20.22%	20.21%	0.43	No trend
South	38.3%	41.29%	38.15%	37.39%	39.51%	41.81%	41.88%	0.42	No trend
West	10.78%	9.46%	10.46%	11.21%	10.99%	10.31%	10.52%	0.97	No trend
Teaching status and location									
Rural	6.12%	5.75%	4.59%	4.19%	3.61%	4.48%	4.3%	0.06	No trend
Urban non-teaching	29.97%	31.45%	30.01%	29.01%	18.92%	20.19%	15.62%	<0.001	Decreasing
Urban teaching	63.9%	62.79%	65.39%	66.78%	77.45%	75.31%	80.01%	<0.001	Increasing
Hospital bed size									
Small	11.86%	8.95%	7.34%	12.57%	17.09%	18.01%	20.93%	<0.001	Increasing
Medium	26.37%	22.62%	23.3%	29.48%	28.29%	29.8%	29.51%	0.07	No trend
Large	61.76%	68.42%	69.35%	57.93%	54.61%	52.18%	49.55%	<0.001	Decreasing
Disposition									
Regular	79.01%	80.44%	79.28%	78.3%	77.61%	76.33%	74.51%	<0.001	Decreasing
NF	10.34%	9.14%	9.8%	10.32%	10.54%	10.87%	11.28%	0.002	Increasing
Home health	2.36%	2.45%	3.27%	3.76%	4.35%	4.32%	4.76%	<0.001	Increasing
AMA	8.26%	7.96%	7.6%	7.6%	7.48%	8.46%	9.42%	0.03	Increasing
*Mean total Charge ($)	28,016	26,459	30,181	33,863	37,748	40,932	43,106	<0.001	Increasing
Financial burden ($)	10.8 billion	7.96 billion	9.5 billion	9.19 billion	10.3 billion	14.8 billion	19 billion	<0.001	Increasing
*Mean LOS (days)	5.84	5.27	5.29	5.27	5.44	5.46	5.47	0.42	No trend

System based side-effects

All the systemic comorbidities increased from 2006 to 2018. This data is shown in Table 2.

**Table 2:** System based side effects HTN=Hypertension; PVD=Peripheral vascular disease; HIV=Human immunodeficiency virus; MI=Acute myocardial infarction; Hepatitis=Hepatitis B/C

Comorbidity	2006	2008	2010	2012	2014	2016	2018	p-Trend	Trend Direction
Cardiovascular									
MI	4.13%	4.79%	5.62%	6.44%	6.83%	6.99%	7.85%	<0.001	Increasing
HTN uncomplicated	23.69%	26.53%	31.14%	32.43%	33.7%	32.05%	27.55%	<0.001	Increasing
HTN complicated	5.51%	6.61%	7.93%	8.78%	9.92%	11.07%	17.53%	<0.001	Increasing
Arrhythmia	6.49%	7.5%	8.74%	10.5%	11.66%	10.53%	12.48%	<0.001	Increasing
Cardiomyopathy	0.25%	0.28%	0.19%	0.25%	0.2%	0.43%	0.5%	<0.001	Increasing
Heart failure	7.51%	7.66%	8.65%	9.77%	11.2%	13.15%	15.13%	<0.001	Increasing
PVD	0.8%	0.91%	1.27%	1.44%	1.84%	3.74%	3.68%	<0.001	Increasing
Neurological									
Cerebrovascular disease	2.88%	3.34%	3.87%	4.22%	4.49%	4.5%	4.62%	<0.001	Increasing
Hemiplegia/Paraplegia	0.76%	0.91%	1.19%	1.25%	1.3%	1.8%	1.85%	<0.001	Increasing
Seizure	0.06%	0.2%	0.27%	0.29%	0.28%	0.16%	0.25%	<0.001	Increasing
Pulmonary									
Asthma	11.63%	11.91%	13.28%	13.73%	14.5%	13.56%	10.62%	0.86	No trend
Infectious disease									
HIV	4.56%	4.28%	4.57%	3.72%	3.52%	3.02%	2.89%	<0.001	Decreasing
Hepatitis	11.09%	12.29%	13.3%	14.69%	15.22%	15.3%	14.66%	<0.001	Increasing
Psychiatry									
Psychosis	14.17%	13.5%	14.69%	15.7%	16.92%	16.76%	16.69%	<0.001	Increasing
Depression	15.02%	16.43%	16.63%	18.46%	20.04%	29.24%	29.25%	<0.001	Increasing

Cardiovascular

There is an increase in prevalence of acute myocardial infarction (4.13 to 7.85%), uncomplicated hypertension (23.69% to 27.55%), hypertension urgency/emergency (5.51% to 17.33%), arrhythmias (6.49% to 12.48%), non-ischemic cardiomyopathy (0.25% to 0.5%), heart failure (7.51% to 15.13%), and peripheral vascular disease (0.8% to 3.68%) since 2006 with p<0.001.

Neurological

The prevalence of cocaine associated cerebrovascular disease (2.88% to 4.62%), hemiplegia/paraplegia (0.76% to 1.85%) and seizure (0.06% to 0.25%) has increased from 2006 with p<0.001.

Infectious Diseases

Human immunodeficiency virus (HIV) prevalence has decreased from 4.56% in 2006 to 2.89% in 2018, while hepatitis B/C had increased (11.9% to 15.3%) with p<0.001.

Psychiatry

There has been an increase in the prevalence of psychiatric disorders over the past decade, with psychosis (14.17% to 16.69%) and depression (15.02% to 29.25%) rising to almost twice as in 2006.

Top ten reasons for hospital admission

Major depressive disorder was the most common reason for hospital admission, followed by cocaine poisoning and schizoaffective disorder. Other causes like alcohol withdrawal, mood disorders, and sepsis also contributed significantly towards hospital admission. This data has been described in Table 3.

**Table 3:** Top 10 reasons for hospital admission

1. Major depressive disorder with psychotic symptoms
2. Cocaine poisoning
3. Schizoaffective disorder, bipolar type
4. Alcohol dependency with withdrawal
5. Drug induced mood disorder
6. Sepsis
7. Bipolar disorder
8. Chest pain
9. Cocaine dependency with withdrawal
10. Heart Failure

Concomitant drug abuse and in-hospital mortality

**Figure 2:**
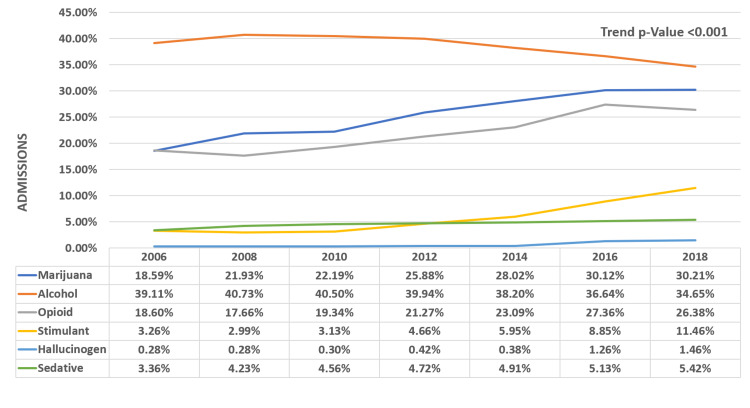
Concomitant drug abuse in admitted patients with cocaine use

Alcohol is the most abused drug along with cocaine; the percentage use decreased (39.11% to 34.65%) with p<0.001, while marijuana use (18.59% to 30.21%) and opioid use (18.6% to 26.38%) has been increasing since 2006 (p<0.001). Use of concomitant stimulants, hallucinogens, and sedatives is also growing, as shown in Figure 2.

Adjusted in-hospital mortality was higher with concomitant use of the stimulants, with an adjusted odds ratio of 1.28 (CI 1.02 -1.6, p=0.03). Adjusted In-hospital mortality for most common concurrent drugs used is presented in Table 4.

**Table 4:** In-hospital mortality when co-ingested with other drugs ^1^ Adj for Age, sex, race, charlson comorbidity index, hospital bed size, teaching status, location and insurance

OUTCOMES	Cocaine (787,118)	Cocaine + Alcohol (1,294,448)	p-Value
In-hospital mortality	1.36%	1.07%	<0.001
	Adjusted odds ratio^1^ 0.99 (CI 0.91-1.08)		0.87

OUTCOMES	Cocaine (787,118)	Cocaine + Opioid (1,015,389)	p-Value
In-hospital mortality	1.36%	1.23%	0.03
	Adjusted odds ratio^1^ 1.11 (CI 0.99-1.23)		0.053

OUTCOMES	Cocaine (787,118)	Cocaine + Stimulant (822,775)	p-Value
In-hospital mortality	1.36%	1.35%	0.92
	Adjusted odds ratio^1^ 1.28 (CI 1.02-1.6)		0.03

OUTCOMES	Cocaine (787,118)	Cocaine + Marijuana (1,017,937)	p-Value
In-hospital mortality	1.36%	0.7%	<0.001
	Adjusted odds ratio^1^ 0.66 (CI 0.58-0.76)		<0.001

OUTCOMES	Cocaine (787,118)	Cocaine + Hallucinogen (789,454)	p-Value
In-hospital mortality	1.36%	0.64%	0.17
	Adjusted odds ratio^1^ 0.68 (CI 0.22-2.15)		0.52

OUTCOMES	Cocaine (787,118)	Cocaine + Sedative (799,184)	p-Value
In-hospital mortality	1.36%	0.9%	0.04
	Adjusted odds ratio^1^ 0.96 (CI 0.62-1.5)		0.88

Financial burden on healthcare and resource utilization

Adjusted total hospitalization charges increased from 2006 to 2018 (28,016 to 43,106, p<0.001) while adjusted mean LOS remained stable (5.84 days to 5.47 days, p=0.42). The total financial burden incurred due to cocaine use-related hospitalizations nearly doubled from ($10.8 billion to $19 billion, p<0.001) (Figure 1).

## Discussion

Demographic trends

Our study showed that the prevalence of cocaine use amongst hospitalized patients in 2018 was 11,891 per million admissions, which has been on the rise since 2014 after being on a decline since 2006. Mustaquim et al. showed that the prevalence of cocaine use in the general population follows the same trend that we saw in our study [12]. During this period, older patients (age>50 years) showed an uptrend in cocaine use while the middle-aged population (age 30-49 years) showed a downtrend. The highest contributors to the prevalence of cocaine use were the middle and older aged population as they have a higher prevalence of depression and other psychiatric conditions. Goldstein et al., in their study, also found that cocaine abuse is increasing in the elderly patients [7]. Contrary to this, a study done by Mustaquim et al. showed the younger population was the highest contributor [12]. However, the study’s major limitation was that the data used was self-reported use of cocaine which raises concerns of recall and social desirability bias. Therefore, strategies should be tailored to this population group for early screening of abuse disorder and early intervention in diagnosed patients to prevent health-related complications. Interventions in cocaine abusers are mainly psychosocial as there is no approved pharmacological treatment for the same [13].

We found an increasing trend of cocaine use in the male sex and all ethnicities except for the Native American population, similar to Mustaquim et al [12]. In their study, Cano et al. state that black, older, and less educated individuals contributed to the cocaine-related overdose deaths [14]. Our study also showed a significant increase in cocaine use among patients treated in small, bedded hospitals and a significant decrease in larger hospitals. These findings suggest that the local availability of illicit substances and pattern of use in the community play a huge role in the prevalence of cocaine abusers, and therefore, these factors should be kept in mind while planning prevention and response strategies [12].

Clinical implications

The most common causes of hospitalization in our study were Major Depressive Disorder (MDD), followed by Cocaine poisoning and schizoaffective disorder. These trends can be attributed to the effects of long-term cocaine use on the pre-frontal cortex and cerebral blood flow, which ultimately affect the mood and cognitive functioning of the brain [15]. Other less common causes included alcohol dependency with withdrawal, drug-induced mood disorders, sepsis, and chest pain. Repeated use of needles and syringes and unhygienic living conditions could put these patients at high risk of contracting bacterial infections leading to sepsis.

Trends of cardiovascular disease

There have been multiple reports of cocaine-induced myocardial infarctions (MI) in patients abusing it [16-18]. It was evident in our study that the prevalence of acute MI increased steadily over the past decade, 4.13 in 2006 to 7.85 in 2018 (p<0.001). Animal models have shown that cocaine use is associated with wall motion abnormalities, ventricular wall dilation, decreased contractility and increased end-diastolic pressure, ultimately leading to cardiomyopathies and heart failure. Cocaine affects blood pressure and causes vasoconstriction by inhibiting preterminal catecholamine reuptake and it also promotes release of endothelin-1 and inhibits release of nitric oxide [16]. Cocaine also causes arrhythmias due to direct and indirect effects on the cardiovascular system [16, 19]. We found a rising prevalence of hypertension, arrhythmias, heart failure, non-ischemic cardiomyopathies, and peripheral vascular diseases in our study. This could be due to the increasing prevalence of cocaine use among the older population who are likely to have obesity, hyperlipidemia, diabetes, etc., which affects the cardiovascular health of patients [20].

Trends of neuropsychiatric disease

Cocaine affects the nervous system by decreasing the seizure threshold [21], cerebral vasospasm, and hypertensive surges [22] and is known to cause seizures, stroke, and psychiatric problems in cocaine users [23, 24]. Our study showed that cocaine use-associated cerebrovascular diseases like stroke, hemiplegia/paraplegia, and seizures are rising. We also found that cocaine use-related psychosis and depressive disorders have increased significantly. These findings were congruent to the study findings of Mustaquim et al [12]. As mentioned earlier, MDD and Schizoaffective disorders were two of the top three causes of hospitalization in cocaine abusers.

Trends of infectious disease

In contrast to previous studies which showed that cocaine users and crack smokers are at high risk of HIV infection, our study revealed decreasing prevalence of HIV [25]. Prevalence of hepatitis B and C has been increasing among these patients. This could be explained by intravenous injections, which is the third most common route of cocaine consumption amongst abusers after snorting and smoking [26]. However, there have been reports of contracting Hepatitis C by sharing straws used for snorting cocaine [27]. In our study, we were unable to differentiate various methods of cocaine use. The decrease in HIV prevalence is promising and indicates that primary and secondary preventive measures are beneficial. This provides further opportunity for a researcher to investigate the factors that could impact the transmission of blood-borne diseases in these high-risk patients. 

Trends of concomitant drug abuse and mortality outcomes

There has been an increasing trend of using other illicit drugs along with cocaine. Multiple studies that show that drug abusers use two or more drugs to get an additive effect of euphoria and nullify the side effects of either drug [26, 28]. Our study showed that there has been a rising trend of concomitant use of marijuana, opioid, other stimulants like methamphetamine, hallucinogens, and other sedatives. These findings are supported by studies done by Narvaez et al., who found similar concomitant use of other drugs amongst cocaine users [28]. However, alcohol use remains the highest, and the trend seems to be decreasing in this population. Concomitant use of these illicit drugs also explains the increasing trend of mood and behavior disorders we found in our study [28]. The mortality rate with concomitant drug use was significantly high with other stimulant use. We did not find a significant difference in mortality rates with alcohol, opioids, sedatives, and hallucinogens. The morbidity, however, has shown concerning trends over the past decade or so. It is linked to various mental health issues, including substance abuse, domestic abuse, and poor health. It is also known to have negative effects on work productivity and social engagement [29, 30]. This in-turn, increases the burden on healthcare utilization and cost.

Strengths and limitations

A major strength of the study was the sample size (261.38 million patients) and duration (12 years). Using the largest publicly available database, we were able to identify patterns in the hospitalizations of patients over a long period.

Our study comes with limitations of a cross-sectional study. In addition, as this study is based on a database, coding errors are possible, and diseases not entered might have been missed. The NIS does not have information regarding emergency department visits, lab values, method of cocaine use and imaging. Patients admitted to the hospital who were never screened for drug abuse would not be included. The income percentile represented is based on the patient’s residence zip code, and it might not truly represent the actual financial status of the patient.

## Conclusions

Substance use disorder is one of the modern pandemics and contributes to a significant burden on healthcare. This retrospective study from the National Sample Database focused on evaluating the trends related to the use of cocaine in hospitalized patients. The study reveals a recent uptrend in cocaine use among hospital admissions in the US from 2006 to 2018. Cocaine use is associated with a high prevalence and increasing rates of systemic manifestations with a negative impact on numerous systems. Oddly enough, the study showed that although hepatitis rates are growing, HIV rates are on a downward decline. The most common reason for hospital admission in those with cocaine use was Major depressive disorder. Alcohol, marijuana, and opioids are the most common drugs abused, along with cocaine. The use of marijuana and opioids is increasing over the years. The financial burden incurred due to cocaine use-related hospitalizations nearly doubled from 2006 to 2018 ($10.8 billion vs. $19 billion, p<0.001). Cocaine use is an alarming problem in the United States and impacts multiple aspects of patients’ welfare and outcome. Further studies to identify the cause of the recent uptrend, various interventions, and policy measures are required.

## Appendices

**Table 5:** Supplementary table 1

Variable	ICD-9 CM codes	ICD-10 CM codes
Cocaine abuse, dependency, unspecified, poisoning	304.2X (except 304.23) 305.6X (except 305.63) 97081	F14.XX (except F14.11, F14.21) T40.5X, R78.2
Alcohol abuse, dependency, unspecified, poisoning	291.XX, 303.XX (except 303.03) 305.0X (except 305.03), 980	F10.XX (except F10.11, F10.21) Y90, R780
Opioid abuse, dependency, unspecified, poisoning	304.0X (except 304.03) 305.5X (except 305.53)	F11.XX (except F11.11, F11.21) R781, T40.0X, T40.1X, T40.2X, T40.4X
Cannabis abuse, dependency, unspecified, poisoning	304.3X (except 304.33) 305.2X (except 305.23)	F12.XX (except F12.11, F12.21) T40.7X
Stimulant abuse, dependency, unspecified, poisoning	304.4X (except 304.43) 305.7X (except 305.73)	F15.XX (except F15.11, F15.21)
Hallucinogen abuse, dependency, unspecified, poisoning	304.5X (except 304.53) 305.3X (except 305.33)	F16.XX (except F16.11, F16.21) T40.8X, T40.9X
Sedative abuse, dependency, unspecified, poisoning	304.1X (except 304.13) 305.4X (except 305.43)	F13.XX (except F13.11, F13.21)
Acute MI	410.x, 412.x	I21.x, I22.x, I25.2
HTN uncomplicated	401.1, 401.9	I10.x
HTN complicated	402.10, 402.90, 404.10, 404.90, 405.1, 405.9	I11.x-I13.x, I15.x
Arrhythmia	426.10, 426.11, 426.13, 426.2-426.53, 426.6-426.8, 427.0, 427.2, 427.31, 427.60, 427.9, 785.0, V45.0, V53.3	I44.1-I44.3, I45.6, I45.9, I47.x-I49.x, ROO.O, ROO.1, ROO.8, T82.1, Z45.0, Z95.0
Cardiomyopathy	425.9	I42.7
Congestive heart failure	428.x	I09.9,I11.0, I13.0, I13.2, I25.5, I42.0, I42.5-I42.9, I43.x, I50.x, P29.0
Peripheral vascular disease	G45.x, G46.x, H34.0, I60.x-I69.x	I70.x, I71.x, I73.1, I73.8, I73.9, I77.1, I79.0, I79.2, K55.1, K55.8, K55.9, Z95.8, Z95.9
Cerebrovascular disease	430.x-438.x	G45.x, G46.x, H34.0, I60.x-I69.x
Hemiplegia/paraplegia	344.1, 342.x	G04.1, G11.4, G80.1, G80.2, G81.x, G82.x, G83.0-G83.4, G83.9
Seizure	3450, 3451	G405
Asthma	493.XX	J45.XX
HIV	042.x-044.x	B20.x-B22.x, B24.x
Hepatitis B/C	070.XX	B16.XX, B17.XX, B18.XX, B19.XX
Psychosis	295.x-298.x, 299.1	F20.x, F22.x-F25.x, F28.x, F29.x, F30.2, F31.2, F31.5
Depression	300.4, 301.12, 309.0, 309.1, 311	F20.4, F31.3-F31.5, F32.x, F33.x, F34.1, F41.2, F43.2
